# Lumpy skin disease diagnosis in cattle: A deep learning approach optimized with RMSProp and MobileNetV2

**DOI:** 10.1371/journal.pone.0302862

**Published:** 2024-08-05

**Authors:** Sheikh Muhammad Saqib, Muhammad Iqbal, Mohamed Tahar Ben Othman, Tariq Shahazad, Yazeed Yasin Ghadi, Sulaiman Al-Amro, Tehseen Mazhar

**Affiliations:** 1 Institute of Computing and Information Technology, Gomal University, Dera Ismail Khan, Pakistan; 2 Department of Computer Science, College of Computer, Qassim University, Buraydah, Saudi Arabia; 3 Department of Computer Science, COMSATS University Islamabad, Sahiwal, Pakistan; 4 Department of Computer Science and Software Engineering, Al Ain University, Abu Dhabi, United Arab Emirates; 5 Department of Computer Science, Virtual University of Pakistan, Lahore, Pakistan; Beni Suef University Faculty of Veterinary Medicine, EGYPT

## Abstract

Lumpy skin disease (LSD) is a critical problem for cattle populations, affecting both individual cows and the entire herd. Given cattle’s critical role in meeting human needs, effective management of this disease is essential to prevent significant losses. The study proposes a deep learning approach using the MobileNetV2 model and the RMSprop optimizer to address this challenge. Tests on a dataset of healthy and lumpy cattle images show an impressive accuracy of 95%, outperforming existing benchmarks by 4–10%. These results underline the potential of the proposed methodology to revolutionize the diagnosis and management of skin diseases in cattle farming. Researchers and graduate students are the audience for our paper.

## 1. Introduction

Lumpy skin disease, which is caused by LSDV [1], poses a significant threat to cattle and can be transmitted by various blood-feeding vectors such as ticks, flies and mosquitoes. Even without prior exposure, affected animals can develop skin nodules, develop a fever and tragically succumb to the disease. Preventive strategies such as vaccination and caring for affected animals remain crucial in the management of this painful disease [2].

Lumpy skin disease is a highly contagious and serious disease that affects a large proportion of cows. Its transmissibility raises concerns about the cross-border transmission that could affect neighbouring countries. The profound impact of the disease on international trade restrictions and reduced agricultural production led to significant economic setbacks. The spread of the disease is closely linked to warm and humid climatic conditions, which favour an increase in populations of biting arthropods [3, 4]. A typical approach by cattle farmers involves a series of steps to treat lumpy skin disease. Initially, traditional remedies are used, including the application of natural leaf extracts and local burns, before seeking the help of veterinarians. However, this process is time-consuming and often leads to a delayed diagnosis of the disease [5, 6].

To better understand the complicated mechanisms underlying lumpy skin disease, a thorough evaluation of deep learning algorithms is performed in the current study. The proposed work does not directly use a convolutional neural network (CNN) model to effectively mitigate the various drawbacks associated with CNNs, including, but not limited to, high computational requirements, significant data labeling requirements, substantial memory consumption, interpretability challenges, limited applicability to sequential data tasks, relatively slow processing, and long training times [7, 8]. However, the use of transfer learning is preferable.

Furthermore, the proposed research introduces the novel integration of MobileNetV2 with the customization of RMSprop to address crucial challenges in the diagnosis of lumpy skin diseases. By mitigating the adverse effects of fading gradients, promoting effective feature reuse, and optimizing parameter utilization, the study aims to revolutionize the effectiveness and efficiency of deep learning models in the diagnosis and treatment of this debilitating disease. The research aims to significantly increase the diagnostic accuracy and treatment efficacy with this proposed approach, thus achieving significant advances in the treatment and control of lumpy skin disease. The research contributions in this thesis are as follows:

- Preparation of a well-balanced training and test dataset with appropriate image distributions.
- Implementation of a Model Utilizing MobileNetV2 Architecture with the RMSprop Optimizer.
- Assessment and comparison of the performance of the MobileRMSNet with other relevant studies.

The rest of the manuscript is organized as follows: The literature review is contained in Section 2. Section 3 describes the proposed model, MobileRMSNet, with a detailed mathematical explanation and visual results. Section 4 discusses the simulation setup of MobileRMSNet and presents the experimental results. Section 5 presents a comparative analysis of the proposed model. Finally, Section 6 presents the conclusion and future work.

## 2. Related works

Most disease research has been conducted using machine learning and deep learning techniques. The literature first looks at machine learning, followed by research into deep learning methods. One study by researchers presented a computerized cataract classification system that relies on fundus images and is documented in [8]. In this study, features were taken from the optic disk and cup. The in vivo Automatic Nuclear Cataract Detection and Classification System has been given the DRISHTI and RIM-ONE V3 fundus picture datasets to find glaucoma [9]. Further, a machine learning and ultrasonography-based automated in vivo nuclear cataract detection and categorization system was created. The following description provides more detail about this method [10]. Subsequently, in [11], the researchers employed an SVM classifier to categorize fundus images as cataract images using an RBF network to assess the severity of the disease and achieved a specificity of 93.33%. In another study [12], a CNN model for automatic glaucoma classification was developed using transfer learning techniques on the DRISHTI and RIM-ONE V3 datasets with fundus images. In addition, a cataract detection method based on an Android smartphone was presented in [13], which used a single-layer perceptron technique that achieved a classification accuracy of 85%.

A considerable amount of research has been devoted to the study of bovine nodular skin disease. Yet, there is a notable lack of technical studies in the field of computer vision that specifically address bovine lumpy skin disease. Although some theoretical studies have been documented, empirical research remains relatively limited. In particular, an evaluation has been outlined that focuses on the qualitative assessment of lumpy skin disease transmission [14–18], using probabilistic methods to assess the associated risks [19, 20]. Recent advances in machine vision and machine learning have driven the development of various methods for human health disease segmentation and classification [21]. For example, an automated method for lung cancer classification, involving a two-step segmentation and classification process, has been introduced based on both classical and transfer learning from chest X-ray images. In particular, including feature selection techniques has helped optimize detection results, leading to a remarkable accuracy rate of over 90% in all disease categories [22].

The literature also covers the various attempts to classify skin lesions in human skin cancer [23]. In this particular instance, high-pass filters to enhance edges and homomorphic filters to lessen lighting effects have been prioritized [24]. Such research highlights the critical function of segmentation and illuminates several cancer features [25]. This feature collection covers form, delineation, asymmetry, and irregularity evaluations. Lesion boundary definition techniques combining weight-based feature selection and morphological filtering have also succeeded [26]. Numerous classification techniques are researched, including deep learning models, decision trees [27], support vector machines (SVMs) [28], and K-nearest neighbours (KNNs) [29–31].

In particular, studies have emphasised the application of GoogLeNet and Inception V3 CNN models for skin cancer classification and the application of the AlexNet model for pattern recognition [32, 33]. In addition, the use of a Deep Full-Resolution Convolution Network (DFRCN) with a SoftMax layer for robust classification purposes was highlighted [34, 35]. Several works were found to rely on conventional machine learning approaches for the study of animal diseases, with a limited number of works utilising the transfer learning method of denseNet121 instead of mobileNet methods [36–38]. Consequently, there are still some challenges, such as improving model accuracy while addressing issues related to model complexity by reducing the number of training parameters, layers, depth, runtime and overall size of the model [39].

## 3. Proposed model: MobileRMSNet

**Fig 1:**
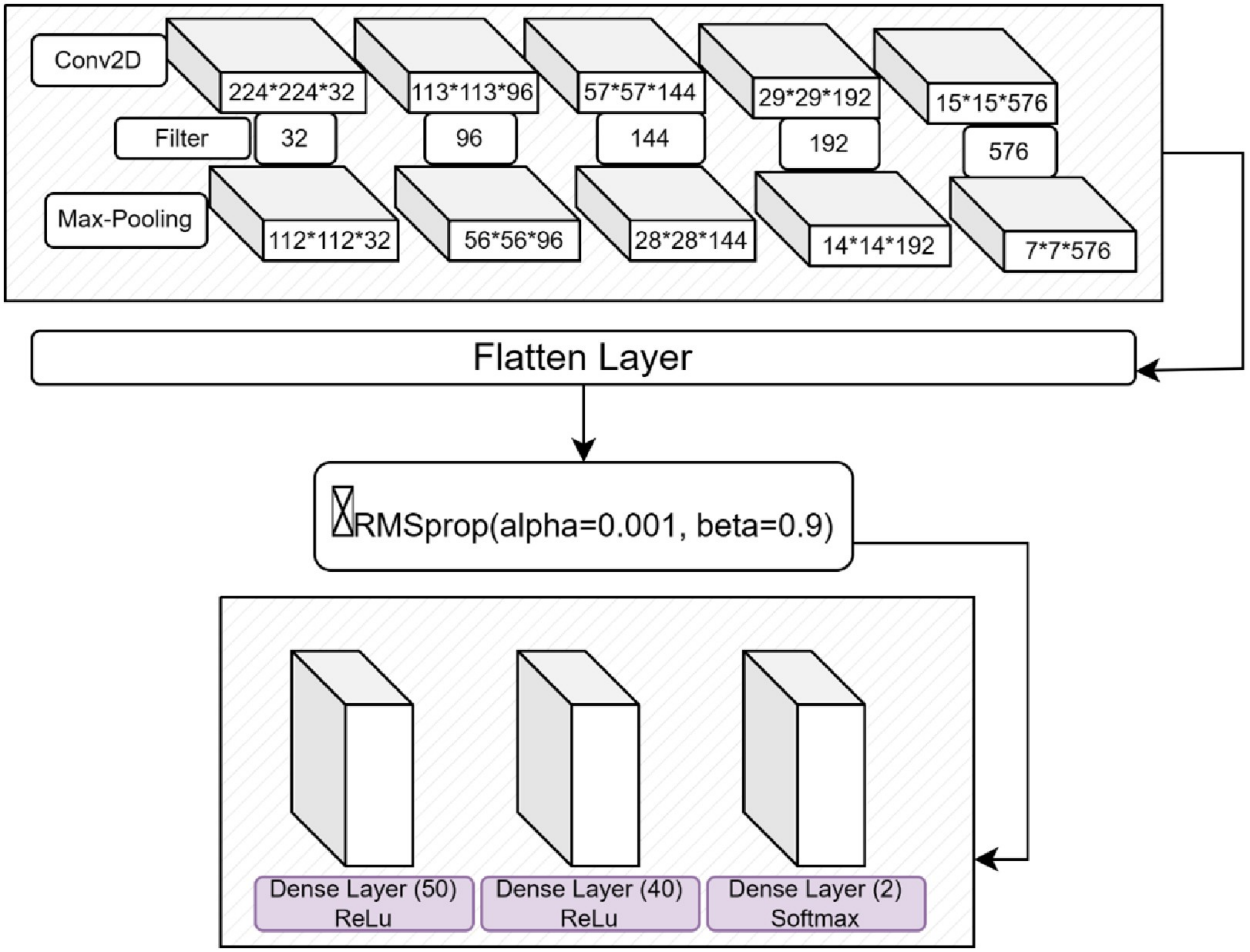
MobileRMSNet for cow disease.

The main goals of integrating MobileNetV2 with the RMSprop optimizer into our model revolve around removing obstacles such as mitigating gradient descent, promoting feature reuse, and minimizing parameter consumption. These goals significantly improve the training process of our deep learning model. Fig 1 illutrates the proposed Model.

### 3.1. Layers in proposed model

Fig 1 illustrates the structure of a convolutional neural network (CNN) tailored to image classification. The model starts with a Conv2D layer that performs a 2D convolution on the input image and produces a 224x224 output containing 32 feature maps. These feature maps capture different patterns and features within the image. A max-pooling layer is then used to reduce the spatial dimensions to 112x112 while retaining the 32 feature maps to reduce the data size and extract key information.

The image will then have a second Conv2D layer added to it, producing 96 feature maps. This layer pulls even more complex patterns and features from the input image. A max-pooling layer is applied, reducing the map’s dimensions to 56x56. This layer maintains the 96 feature maps while doing the downsampling and feature extraction procedures. The pattern repeats itself when the model passes through an increasing number of max-pooling and Conv2D layers; the spatial dimension decreases, and the model’s feature mappings grow bigger with each additional layer.

Using the highest Conv2D layer, 576 feature maps are created to capture the highly complex features in the data. The last max-pooling layer decreases the spatial dimensions to 7x7 while maintaining the 576 feature maps. The most important things won’t change as a result. The multidimensional input is then transformed into a one-dimensional vector by the flattening layer, which prepares it for processing by completely and densely interconnected layers.

Two dense layers follow, the first of which consists of 40 neurons and enables the model to understand complicated patterns and relationships in the data. The last dense layer consists of 2 neurons, indicating the binary classification nature of the model. It uses the softmax function to provide two outputs representing the estimated percentage of healthy cows and cows with lumps, making it well-suited for binary image classification.

### 3.2. RMSprop optimizer

The RMSprop optimizer dampens fluctuations in the vertical, which allows the use of a higher learning rate so that the algorithm can make greater progress in the horizontal, which accelerates convergence. The following equations describe the calculation of the gradients for both RMSprop and gradient descent with momentum. Normally, the momentum value, referred to as β, is set to 0.95, which results in 1- β being 0.005. In this context, the learning rate, represented by α, is specifically set to 0.001. These parameters are chosen to optimize the model fitting process.

When applying this optimizer during backpropagation, the bias β and the weight W are modified by Eqs 1 and 2, where Vdw and Vdb denote specific values derived from Eqs 3 and 4, respectively and are used to minimize the loss. dw denotes the weight derivative, while db represents the prestress derivative concerning the loss. Here, epsilon € denotes a non-zero value that is incorporated to protect the model from significant errors in the case of 0 values for $\sqrt{vdw}$ and $\sqrt{vdb}$.


$$W=W_{t-1}-\alpha .\frac{dw}{\sqrt{vdw}-\varepsilon }$$ (1)

$$b=b_{t-1}-\alpha .\frac{db}{\sqrt{db}-\varepsilon }$$ (2)

$$VDW=\beta .Vdw+(1-\beta ).{\left(dw\right)}^{2}$$ (3)

$$Vdb=\beta .Vdb+(1-\beta ).db^{2}$$ (4)

### 3.3. Softmax-based prediction scheme

By applying softmax as the output layer for the hidden layers, the probability of predicting target labels (e.g., for the cow disease, [1,0] signifies ’healthycow,’ and [0,1] represents ’limpycow’) is computed. The net input is evaluated using a formula in Eq 5, where the weight vector is denoted as ’w,’ the input vector as ’x,’ and the bias as ’b.’ Softmax computation can be performed based on Eq 5, leading to the outcomes illustrated in Eqs 6, 7 and 8.

$$f(z_{i})=\frac{e^{z}i}{\sum_{j}e^{z}j}$$ (5)

Where,$z=(\sum_{i-1}^{n}x_{i}w_{i}+b_{i})$.


$$output=\left\{\begin{array}{ll} 0, & healthycow=0\%\\ 1, & lumpycow=100\% \end{array}\right.$$ (6)

$$output=\left\{\begin{array}{ll} 1, & healthycow=100\%\\ 0, & lumpycow=0\% \end{array}\right.$$ (7)

$$output=\left\{\begin{array}{ll} 0.8, & healthycow=80\%\\ 0.2, & lumpycow=20\% \end{array}\right.$$ (8)

### 3.4. Functionality of model

**Fig 2:**
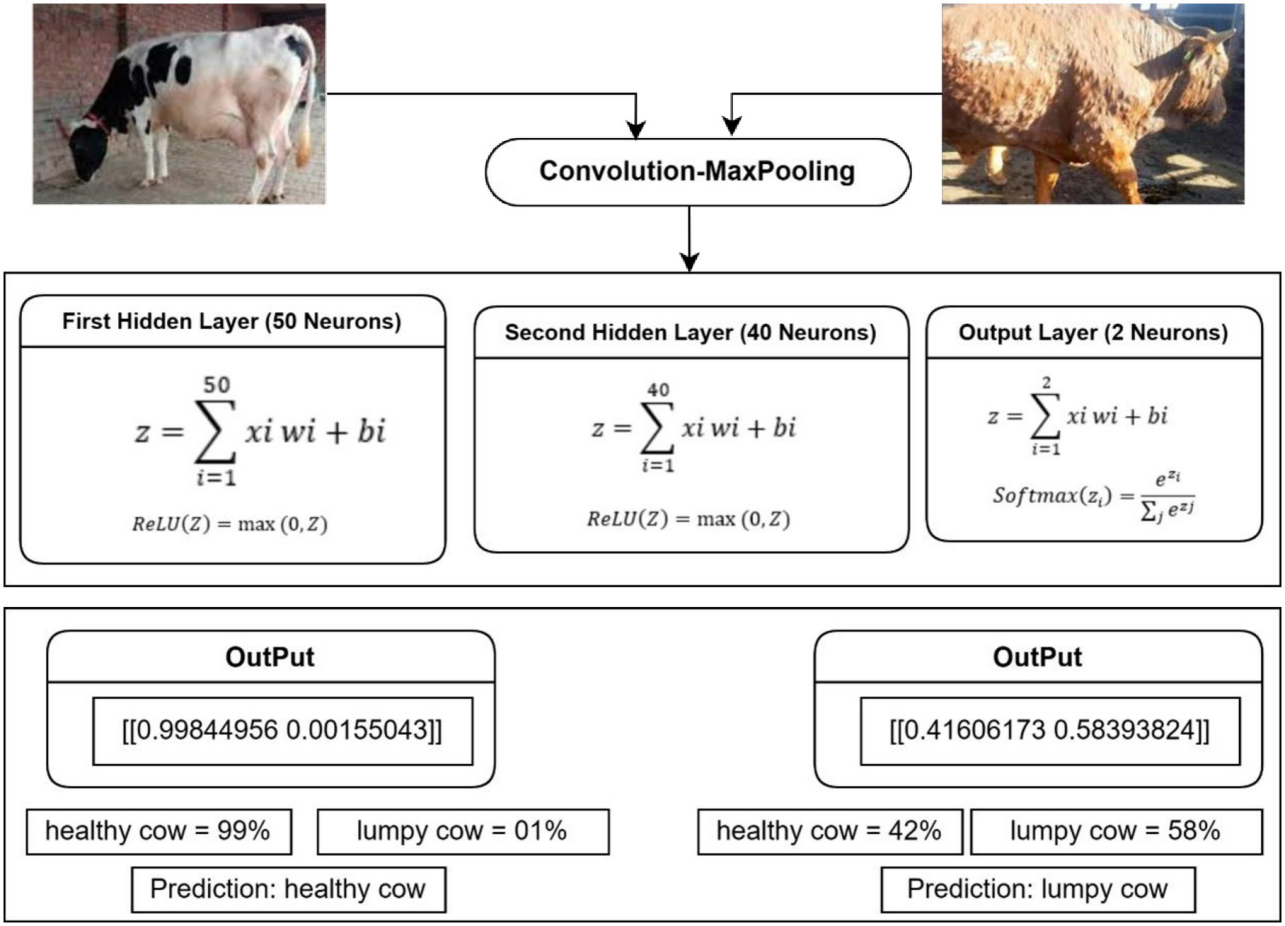
Functionality of the proposed model.

To demonstrate the functionality of the above model, two different images of cows were fed into the model, one showing a ’healthy’ cow and the other a ’lumpy’ cow. Following the MobileNetV1-derived operations of convolution and max-pooling in conjunction with the integration of the RMSprop optimizer and the application of the softmax function on the output layer, the model successfully and accurately classified the images and correctly identified them as representations of ’healthy’ and ’lumpy’ cows, as vividly illustrated in Fig 2.

### 3.5. Simulation setup of MobileRMSNet

The implementation of the proposed model MobileRMSNet was realized by using important Python libraries, in particular, the modules ’MobileNetV2’, ’Dense’, ’Flatten’ and ’Optimizers’ [35-]. Throughout the training process, the model worked with images of dimensions 224x224, with a specified batch size of 32, to ensure efficient handling of the data during the training phase. The model uses 5 epochs to ensure adequate convergence and comprehensive learning and to ensure the best fit of the model to the dataset.

### 3.6. Dataset collection

The dataset encompassed a total of 464 images of healthy cows and 329 images of cows affected by lumpy skin disease. A key challenge was to develop an optimal strategy for partitioning these images to effectively meet the requirements of the model. Through careful consideration of each image, a meticulous process was undertaken that resulted in 334 images being designated for training as representations of healthy cows and 130 images for testing purposes. Similarly, 229 images were selected for training and 100 images for testing to ensure a balanced and comprehensive representation of both healthy and lumpy cows in the training and testing datasets. To ensure diversity and completeness, particular emphasis was placed on including a wide array of images in the training and testing datasets, each of which was diverse and representative. A selection of the training and test datasets is shown in Table 1 and Table 2 respectively.

**Table 1:** Sample data from training dataset.

Class Name	Training Set
Healthy Cow	334
Lump Cow	229

**Table 2:** Sample data from test dataset.

Class Name	Training Set
Healthy Cow	130
Lump Cow	100

### 3.7. Model implementation

**Fig 3:**
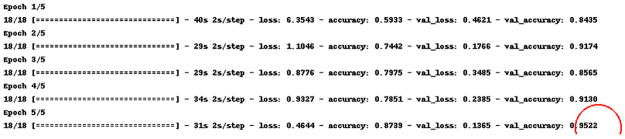
Implementation of model-fitting.

The depiction of MobileRMSNet’s Jupyter file in Fig 3 illustrates a remarkable milestone: in the fifth epoch, a remarkable accuracy of 95% was achieved for the test data. It is worth noting that the time taken during the first five epochs was between 30 and 40 seconds, indicating efficient use of computational resources. Furthermore, a clear trend shows a steady decrease in the loss function over successive epochs for both the training and test data, indicating a continuous improvement in the predictive capabilities and generalization performance of the model.

## 4. Results and discussions

Since the model was trained on the training set, the trained model was applied to the test data. The extracted values on the output layer for all test data were calculated.

In this study, a comprehensive analysis was performed assessing the agreement between the expected and predicted results for the categories "truly healthy cows" and "truly lumpy cows"," represented as binary vectors [1,0] and [0,1], respectively. The softmax values, detailed in Table 3, have a two-component structure where the classification depends on the relative sizes of the two elements. When the first element assumes a higher value, the corresponding vector corresponds to [1,0]; conversely, it is represented as [0,1] when the second element predominates. The final predicted class derived from the values highlighted in Table 3 is detailed in Table 4 and provides a comprehensive overview of the model’s classification performance.

**Table 3:** Values at output layer using softmax.

Name of Image	Softmax Result
healthycows/imgs280.jpg	[[0.6344104–0.3655896]]
healthycows/imgs381.jpg	[[9.9998260e-01 1.7461813e-05]]
healthycows/imgs382.jpg	[[0.92317575 0.07682431]]
healthycows/imgs384.jpg	[[0.98130196 0.01869801]]
healthycows/imgs385.jpg	[[9.999949e-01 5.074137e-06]]
lumpycows/img1044.jpg	[[2.4418184e-04 9.9975580e-01]]
lumpycows/img1045.jpg	[[0.03002201 0.969978]]
lumpycows/img1046.jpg	[[0.05687745 0.9431225]]
lumpycows/img1047.jpg	[[0.06522743 0.93477255]]
lumpycows/img1048.jpg	[[0.0326722 0.96732783]]

**Table 4:** True prediction of classes.

Name of Image	Actual Class	Predicted Class	Confusion Matrix Parameters
healthycows/imgs380.jpg	HC	HC	✓ HC
healthycows/imgs381.jpg	HC	HC	✓ HC
healthycows/imgs382.jpg	HC	HC	✓ HC
healthycows/imgs384.jpg	HC	HC	✓ HC
healthycows/imgs385.jpg	HC	HC	✓ HC
lumpycows/img1044.jpg	LC	LC	✓ LC
lumpycows/img1045.jpg	LC	LC	✓ LC
lumpycows/img1046.jpg	LC	LC	✓ LC
lumpycows/img1047.jpg	LC	LC	✓ LC
lumpycows/img1048.jpg	LC	LC	✓ LC

HC = healthycow LC = lumpycows

✓ HC = True healthycow

✓ LC = true lumpycows

### 4.1. Scientific investigation

In the context of this academic study, Table 5 takes a unique approach by showing the actual images themselves instead of referring exclusively to captions or names. This deliberate methodological choice stems from the intention to increase precision and reduce potential ambiguities that could arise from the exclusive use of image captions. By including the authentic images directly in Table 5, researchers can effectively communicate their findings and facilitate other researchers’ replication of their work. This novel approach significantly increases the transparency and reproducibility of the study and underscores its essential role in maintaining the rigour and credibility of scientific research.

**Table 5:** Visual representation of predicted and actual healthy and lumpy cows.

Actual vector	Softmax Result	Predicted Vector	Found Parameter
[1.0.]	[[0.6344104–0.3655896]]	[1.0,0.0]	✓ HC
[1.0.]	[[9.9998260e-01 1.7461813e-05]]	[1.0,0.0]	✓ HC
[1.0.]	[[0.92317575 0.07682431]]	[1.0,0.0]	✓ HC
[1.0.]	[[0.98130196 0.01869801]]	[1.0,0.0]	✓ HC
[1.0.]	[[9.999949e-01 5.074137e-06]]	[1.0,0.0]	✓ HC
[1.0.]	[[2.4418184e-04 9.9975580e-01]]	[1.0,0.0]	✓ LC
[1.0.]	[[0.03002201 0.969978]]	[1.0,0.0]	✓ LC
[1.0.]	[[0.05687745 0.9431225]]	[1.0,0.0]	✓ LC
[1.0.]	[[0.06522743 0.93477255]]	[1.0,0.0]	✓ LC
[1.0.]	[[0.0326722 0.96732783]]	[1.0,0.0]	✓ LC

✓ HC = True healthycow

✓ LC = true lumpycows

### 4.2. Mistakenly predicted classes

The model showed impressive accuracy in correctly predicting all images of healthy cows. However, there were a few cases where images of lumpy cows were misclassified as ’healthy’ cows, as highlighted in Table 6. In particular, ’image 1034’ and ’image 1122’ were taken from a considerable distance, making it difficult for the model to detect the lumps or patches, resulting in them being classified as ’healthy’ with a confidence level of over 90%.

**Table 6:** Mistakenly predicted class.

Actual vector	PredictIon as healthy	PredictIon as lumpy	Predicted Class
LC	99%	01%	× HC
LC	72%	28%	× HC
LC	96%	04%	× HC
LC	84%	16%	× HC
HC	38%	625	× HC

HC = healthycow LC = lumpycows

✓ HC = True healthycow

✓ LC = true lumpycows

In addition, the ’image1051’ offered a complex scenario, as it contained a cow, text, and a human figure. One cow appeared relatively small in the image, while the other took up a more significant part. The model struggled to accurately calculate the predicted values due to the surrounding context and complexity and eventually labelled this ’lumpy’ image as a ’healthy’ cow with a confidence level of 72%.

The image quality of ’image 1072" was inferior, making it difficult to recognize the lumps or spots. Therefore, this image was also incorrectly classified as a ’healthy’ cow. ’Image 425’ shows the leg of a healthy cow; however, the poor quality of the image led to the black colour being incorrectly interpreted as a lump. Consequently, the model classification for this image resulted in a 38% probability of ’healthy cows’ and a 62% probability of ’lumpy cows.’ These results highlight the importance of using high-quality cameras when using the model and ensuring that there are no humans near the cow, as these factors can significantly affect the model’s accuracy in detecting lumpy skin disease in cows.

## 5. Statistical results

A confusion matrix is a table that visualizes the performance of a classification model. It shows how many predictions the model has made correctly and incorrectly for each class. It is divided into four quadrants, each of which represents a type of prediction error. The four possible outcomes of a binary classification problem are:

- True healthycows (TP): it shows that the “healthycows” classis predicted in correct way by the model
- True lumpycows (TN): it illustrates that the “lumpycows” classis correctly predicted
- False healthycows (FP): it directs that the “healthycows” classis not predicted correctly
- False lumpycows (FN): it indicates that the “lumpycows” class is not predicted by the model correctly

As part of this scientific investigation, MobileRMSNet made predictions about a series of images. From the entire dataset, 127 images were successfully classified as "true healthycows"," while 4 images were incorrectly labeled as "false lumpycows" In addition, the model correctly identified 93 images as "true lumpycows"," but incorrectly assigned 7 images to the "false healthy cows " category.

### 5.1. Precision

Precision tells us how many of the positive guesses were right (true positives). We can calculate it using the formula:

$$\text{P}=\frac{\text{TS}}{TS+FS}$$

In this case, the model achieved 95% precision for ’healthycows’, 96% for ’lumpycows’, and an average precision of 95%.

### 5.2. Recall

Recall tells us how many positive cases the classifier predicted correctly out of all the positive cases in the data. It’s also known as Sensitivity. We calculate it using the formula:

$$\text{R}=\frac{\text{TS}}{TS+FN}$$

In this case, the model achieved 97% recall for ’healthycows’, 93% for ’lumpycows’, and an average recall of 95%.

### 5.3. F1-Score

The F1-Score is a measure that combines both precision and recall. It is often described as the balanced mean of the two. The harmonic mean, used in the F1-Score calculation, is a method for determining an "average" of values, typically considered more suitable for ratios, such as precision and recall, than the regular arithmetic mean. The formula for calculating the F1-Score in this context is

$$\text{F}1=2*\frac{\text{P}*\text{R}}{P+R}$$

In this study, the proposed model achieved a 96% F1-Score for ’healthycows’, 94% for ’lumpycows’, and an average F1-Score of 95%.

### 5.4. Accuracy

The primary metric commonly used for evaluating models is Accuracy, which represents the proportion of correct predictions overall predictions: $Acc = \frac{TS+TNS}{TS+TNS+FS+FNS}$. In this study, the MobileRMSNet achieved 95% accuracy.


$$\text{Acc}=\frac{\text{TS}+\text{TN}}{TS+TN+FS+FN}$$

**Fig 4:**
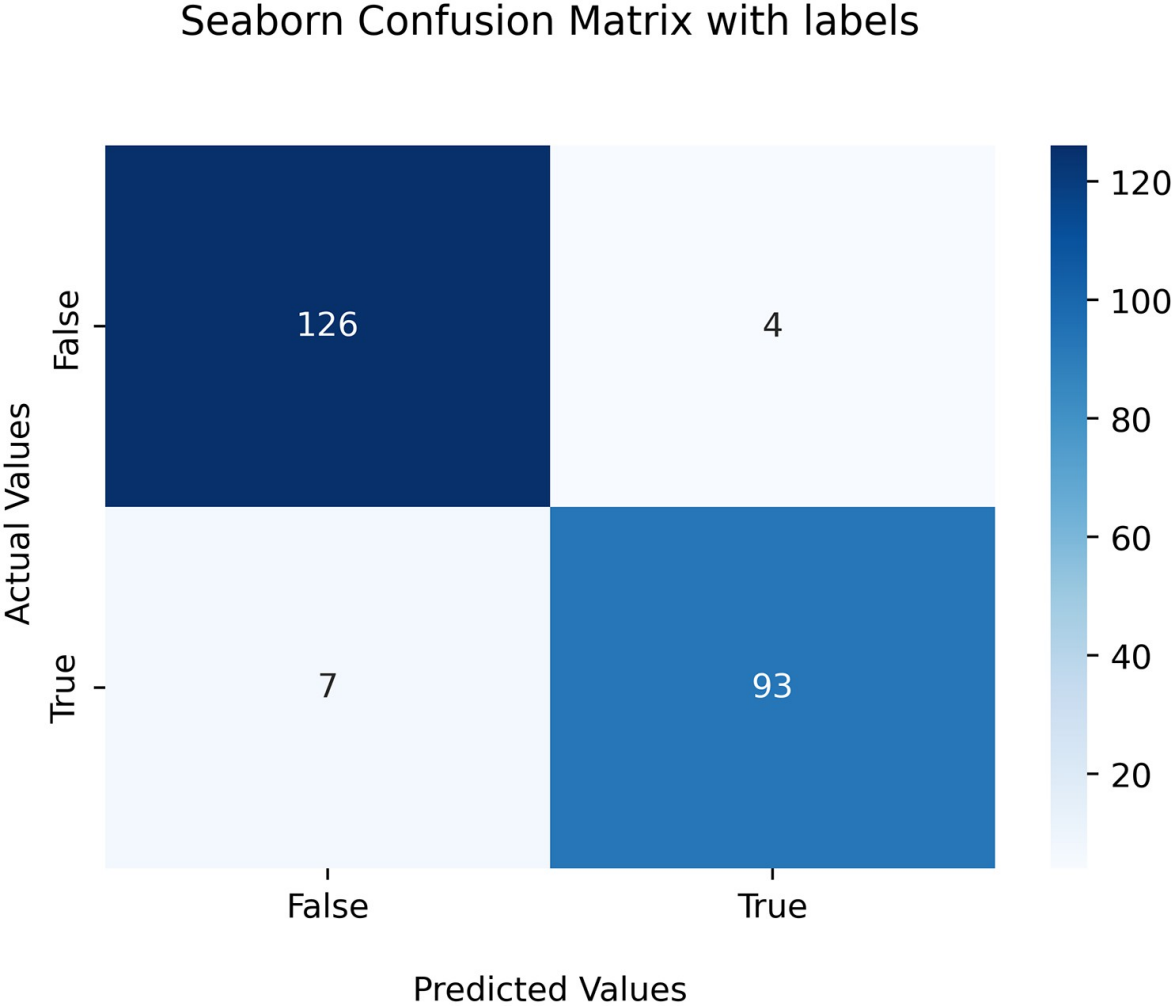
Visual confusion map.

Comprehensive insights into the precision, recall, and accuracy metrics derived from this study are thoroughly documented and delineated in detail within Table 7 and further visually explicated in Fig 4.

**Table 7:** Confusion matrix of the proposed model.

Class	Precision	Recall	F1-Score	Support
‘healthcows’	0.95	0.97	0.96	130
‘lumpcows’	0.96	0.93	0.94	100
Average	0.95	0.95	0.95	230
Accuracy	0.95			

#### 5.4.1. Performance of model

**Fig 5:**
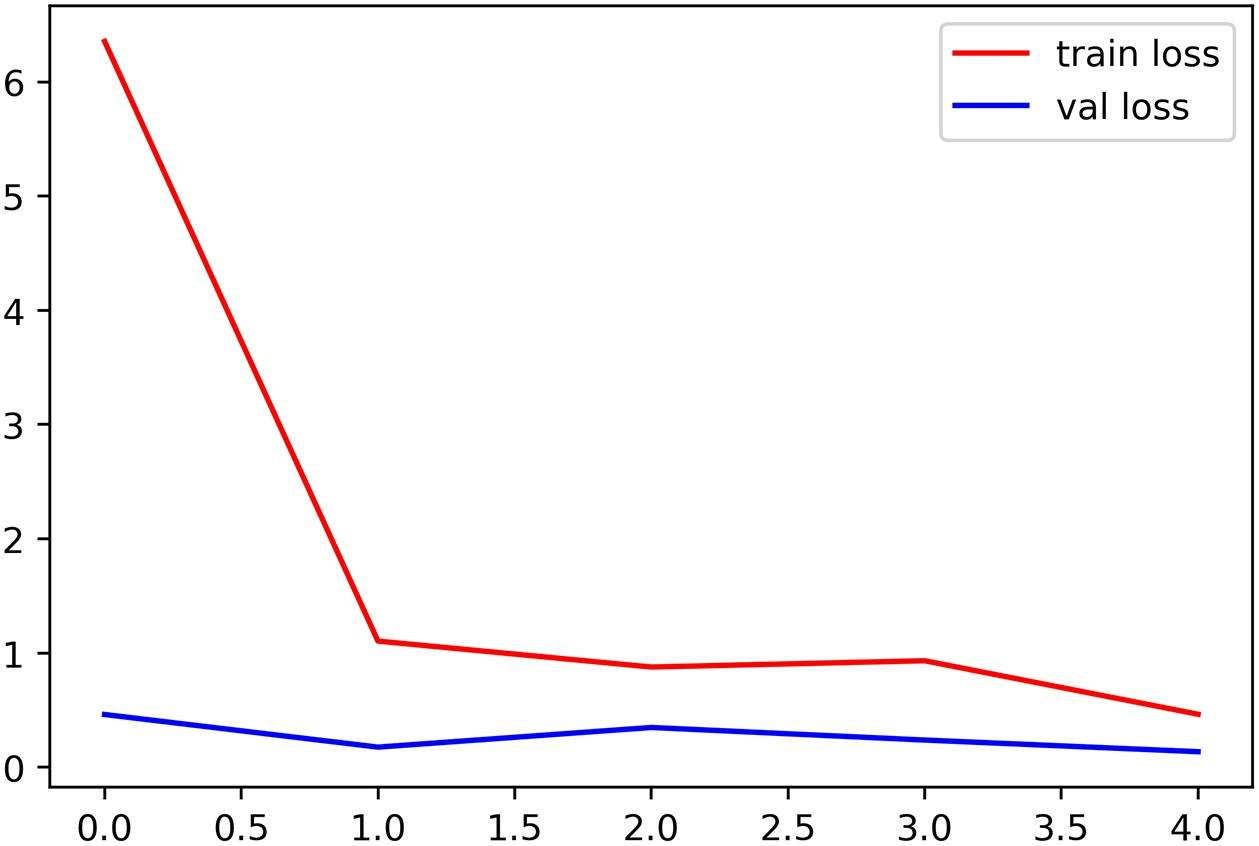
Model performance analysis: Training loss.

The graphical representation in Fig 5 shows a comprehensive performance evaluation of the proposed model, which includes essential metrics such as train loss, test loss, train accuracy, and test accuracy, respectively. In this context, "train loss" refers to the quantification of the deviations or errors of the model from the expected output during the training phase. Conversely, test loss refers to the model’s error rate when working with new, unseen data during the test phase (shown in Fig 5).

**Fig 6:**
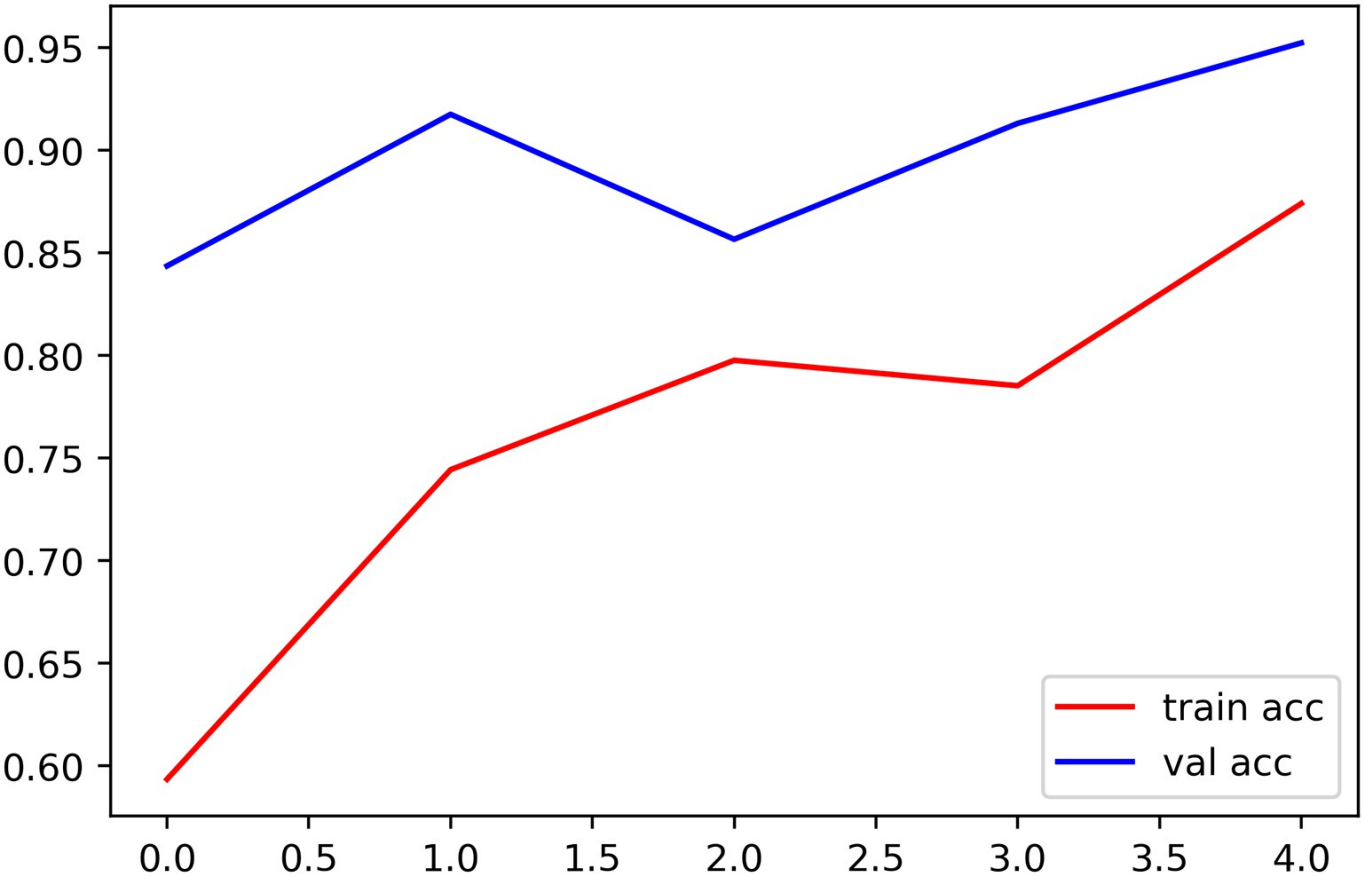
Model performance analysis: Accuracy and loss trends.

Training accuracy’s advantage is that it indicates how successfully the model categorizes the training set. From an alternative perspective, the accuracy of the test provides information about the model’s ability to process new and unexpected data in Fig 6.

A detailed analysis of the patterns and trends in the image shows how well the proposed model performed throughout training and testing. Comparing the train and test losses shows the model’s flexibility and generalization to new data. Studying the link between train and test accuracy may also show the model’s capacity to classify the training and test datasets successfully.

## 6. Discussion

### 6.1. Physical implementation of Mobile RMSNet

As LSD is a critical and perilous disease affecting cows, it not only poses a threat to the afflicted cow but also to all others residing with it. MobileRMSNet primarily focuses on implementing early detection and preventive measures in farms to address this significant concern.

The versatility of the proposed model lies in it being applicable to different categories of farms cited and endemic regions singled out. The way to use it is quite simple: detection is activated either by cameras connected to an application installed on a smartphone or computer. The model is trained, and then it is automatically and directly embedded in the application.

High-resolution cameras are recommended for optimum performance, despite their potential high cost and susceptibility to harm from animals. A practical solution is suggested to mitigate this type of risk by positioning the cameras in elevated locations, such as where cows graze or move in lines, respectively. LSD-affected cattle can be identified through live images captured by the cameras. The owner can be promptly notified via smartphone recognition, enabling swift action to protect the affected cow and prevent the infection from spreading to other cows. This versatile setup can be easily implemented in various endemic farms. Cameras placed in the suggested locations can be covered under shade and positioned at a reasonable height.

At an advanced level, farms can utilize modern technology to improve the detection of LSD-affected cows. This involves the integration of smart cages equipped with sensors and linked to the application. These smart cages, representing potential future developments, can automatically segregate affected cows, offering an additional layer of control and containment. Such an integrated approach underscores the model’s effectiveness in managing and mitigating the impact of LSD in cattle farms.

### 6.2. Comparative analysis of the proposed model

To determine the effectiveness of the suggested model, we compared the results of this study to those of earlier studies in the same field. The primary objective was to assess the model’s capacity to accurately identify and classify particular characteristics, distinguishing between "lumpycows" and "healthy cows" within an extensive collection of cow images. The outcomes clearly showed that during completing this task, the recommended model reached an unusually high degree of accuracy. It emphasizes its remarkable superiority over previous methods in accurately detecting and describing the nuances between the male and female features within the provided image dataset. As shown in Table 8, the performance of the proposed model showed significantly higher accuracy compared to parallel studies in the field.

**Table 8:** Comparison with similar studies.

Model	Accuracy
Ensemble Method	92%
Extreme learning machine (ELM)	90.06%
DenseNet121	89%
CNN-Model	84%
Proposed Work	95%

## 7. Conclusion and future work

Lumpy skin disease, which is caused by the LSD virus, poses a significant threat to cattle and can be transmitted by various blood-feeding vectors such as ticks, flies and mosquitoes. Even without prior exposure, affected animals can develop skin nodules, develop a fever, and tragically succumb to the disease. Preventive strategies such as vaccination-affected animals remain crucial in combating this devastating disease. The proposed methodology (MobileRMSNet) utilises the advanced MobileNetV2 with RMSprop architecture, an innovative deep-learning approach that effectively mitigates the challenges of diagnosing lumpy skin disease. This model represents a significant advance in accurately diagnosing this disease by overcoming complex problems such as fading gradients, feature reuse, and parameter optimization. With a high accuracy rate of 95% and impressive precision and recall rates of 0.95, the proposed methodology far outperforms existing benchmarks. It solidifies its position as a frontrunner in lumpy skin disease diagnosis and management.

In addition to the significant findings, several important limitations have been noted, which will guide further investigation. For instance, further research into images with a resolution of 224 by 224 pixels is required since adjusting this parameter can improve picture quality and optimize feature representation. Changes to this segmentation process could provide a deeper comprehension of the dynamics of the disease. Therefore, we should reconsider how to train and test images by separating them into subgroups of healthy, clumped animals. Investigating other optimization techniques for RMSprop optimization presents a viable means of improving the effectiveness and efficiency of the model’s training operation.

By exploring and implementing optimized strategies for image resizing, data partitioning, and optimizer selection, future studies may improve the accuracy and applicability of MobileRMSNet, paving the way for more comprehensive and refined analyses in lumpy skin disease diagnosis and management.
